# The Prognostic Value of Metabolic Profiling in Older Patients With a Proximal Femoral Fracture

**DOI:** 10.1177/2151459320960091

**Published:** 2020-10-29

**Authors:** Max P.L. van der Sijp, H. Eka D. Suchiman, Monica Eijk, Dina Vojinovic, Arthur H. P. Niggebrugge, Gerard J. Blauw, Wilco P. Achterberg, P. Eline Slagboom

**Affiliations:** 1 4501 Leiden Universitair Medisch Centrum, Leiden, South Holland, Netherlands

**Keywords:** metabolomics, metabolic profiling, biomarkers, prognostics, proximal femoral fracture

## Abstract

**Introduction:**

High mortality rates of approximately 20% within 1 year after treatment are observed for patients with proximal femoral fractures. This preliminary study explores the prognostic value of a previously constructed mortality risk score based on a set of 14 metabolites for the survival and functional recovery in patients with proximal femoral fractures.

**Materials and Methods:**

A prospective observational cohort study was conducted including patients admitted with a proximal femoral fracture. The primary outcome was patient survival, and the recovery of independence in activities of daily living was included as a secondary outcome. The mortality risk score was constructed for each patient and its prognostic value was tested for the whole population.

**Results:**

Data was available form 136 patients. The mean age of all patients was 82.1 years, with a median follow-up of 6 months. Within this period, 19.0% of all patients died and 51.1% recovered to their prefracture level of independence. The mortality score was significantly associated with mortality (HR, 2.74; 95% CI, 1.61-4.66; P < 0.001), but showed only a fair prediction accuracy (AUC = 0.68) and a borderline significant comparison of the mortality score tertile groups in survival analyses (P = 0.049). No decisive associations were found in any of the analyses for the functional recovery of patients.

**Discussion:**

These findings support the previously determined prognostic value of the mortality risk score. However, the independent prognostic value when adjusted for potential confounding factors is yet to be assessed. Also, a risk score constructed for this specific patient population might achieve higher accuracies for the prediction of survival and functional recovery.

**Conclusions:**

A modest prediction accuracy was observed for the mortality risk score in this population. More elaborate studies are needed to validate these findings and develop a tailored model for clinical purposes in this patient population.

## Introduction

Metabolic profiling in epidemiological or clinical cohorts is the simultaneous measurement of numerous metabolites: molecules involved in metabolic processes. Many metabolites have been identified as biomarkers for aspects of health and disease such as mortality, nutritional state and cognitive ability.^1,2^ As such, metabolic profiling may potentially provide an alternative to clinical data for long-term prognostics.^3-6^ A mortality risk score was constructed on the basis of 14 circulating metabolites that were independently associated with mortality in a range of EU population-based cohorts, predicting all-cause 5-10 years mortality.^3^ These 14 metabolites are known to be involved in processes including inflammation, glycolysis, fatty acid and lipoprotein metabolism and fluid balance.^3^

High mortality rates of approximately 20% within 1 year after treatment are observed for patients with proximal femoral fractures.^7-9^ Some of the metabolites included in the mortality risk score have also been studied in patients with a proximal femoral fracture.^10^ Low levels of the plasma protein albumin, considered an important marker of the nutritional status, is associated with adverse outcomes.^1,11-13^ Other markers that have been associated with mortality by multiple studies in patients with proximal femoral fractures include low preoperative hemoglobin levels,^11,14,15^ low total leukocyte count^11,16,17^, high creatinine,^11,14^ high parathyroid hormone,^1,11,18^ high troponins^19^ and high potassium.^14^

Evidence on the value of metabolic profiling for the prognostics of functional recovery in fracture patients is more limited. Only anemia on admission was deemed a relevant prognostic factor with a weak level of evidence in a recent systematic review regarding functional recovery.^20^ Studies on albumin and vitamin D did not present convincing evidence.^20^

Assessments of metabolites associated with the outcomes after a proximal femoral fracture may potentially improve the prognostic accuracy, and further substantiate the metabolomic relevance for patient outcomes.^1^ A pilot study was conducted using a newly constructed cohort of older patients with proximal femoral fractures. This preliminary study explores the prognostic value of a previously constructed mortality risk score based on a set of 14 polar metabolites, lipoproteins, fatty acids and inflammatory proteins for the survival and functional recovery in patients with proximal femoral fractures.

## Methods

### Patients

A single-center prospective observational cohort study included patients with a proximal femoral fracture admitted between December 2019 and May 2020. All patients with pathological fractures, bilateral fractures and less than 18 years of age were excluded.

### Treatment and Assessments

Patients were treated with routine care and data were registered in a coded database by the treating physicians. No individual informed consents were obtained due to the observational nature of the study and the use of routinely collected anonymous data and samples only.

The registered baseline characteristics included age, sex, general health status using the American Society of Anesthesiologists classification,^21^ nutritional status using the Mini Nutritional Assessment—Short Form,^22,23^ prefracture residency (categorized as at home, at home with homecare or a residential home, or a nursing home) and cognitive impairment (defined as any previously diagnosed form of dementia or a Six-item Cognitive Impairment Test ≥11 upon admission). The (prefracture) baseline of independence in activities of daily living (Katz Index of Independence in Activities of Daily Living, Katz ADL)^24^ and mobility (the Parker Mobility Score)^25^ were assessed retrospectively during admission, considering the period directly before the fracture. The fractures were classified as either femoral neck fractures or (sub)trochanteric fractures. Treatment type (osteosynthesis, prosthesis or conservatively) was registered before discharge. Prefracture community dwelling patients were requested for routine outpatient checkups at 6 weeks, 3 months and 1 year after surgery. Prefracture institutionalized patients, or those not attending for any reason, had a checkup by phone, either with the patient, or an (in)formal caregiver.

### Blood Sampling and Metabolic Profiling

Residual blood from the routine venipuncture performed at the emergency department for preoperative blood work (EDTA plasma) was collected and stored for metabolic profiling by an external laboratory (Nightingale Health Ltd., Helsinki, Finland). The method for quantifying the metabolites using high-throughput NMR metabolomics has been described in depth previously.^26^ The method provides simultaneous quantification of routine lipids, lipoprotein subclass profiling with lipid concentrations within subclasses, fatty acid composition, and low molecular metabolites, including amino acids, ketone bodies, and gluconeogenesis related metabolites, in molar concentration units. The technology has regulatory approval (CE) and 37 biomarkers have been clinically certified for diagnostic use.

The obtained set of 272 metabolites includes the 14 used for the mortality risk score: total lipids in chylomicrons and extremely large VLDL (XXL-VLDL-L), total lipids in small HDL (S-HDL-L), mean diameter for VLDL particles (VLDL-D), ratio of polyunsaturated fatty acids to total fatty acids (PUFA/FA), glucose, lactate, histidine, isoleucine, leucine, valine, phenylalanine, acetoacetate, albumin and glycoprotein acetyls.^3^

### Outcomes

The primary outcome of this study was patient survival, defined by the period between surgery (or admission for conservatively treated patients) and death due to any cause within 1 year.

The secondary outcome was the recovery of independence in ADL, which was defined as returning to the individual prefracture level of independence using the Katz ADL score assessed 6 weeks, 3 months and 1 year after treatment. Death due to any cause qualified as not returning to the individual prefracture level of independence, to avoid the otherwise consequential loss to follow-up.

### Statistical Analysis

Descriptive statistics are used to compare the patient characteristics and mean metabolite levels for patients who had and had not died. Means (with standard deviations, SD) are provided for continuous data with a normal distribution, and medians (with interquartile ranges, IQR) for data with a non-normal distribution (Kolmogorov-Smirnov test of p< 0.05).

The mortality risk score as constructed by Deelen et al. using the 14 sampled metabolites was calculated for each individual patient. This requires summing the weighted metabolites after log-transformation and scaling them (Appendix A).^3^ Cox survival analyses were used to assess the association between the mortality risk score and survival, and the mortality risk score’s association with the recovery of independence. The prediction accuracy of the mortality risk score was tested for mortality and the recovery of independence using a receiver operating characteristic (ROC) curve with the area under the curve (AUC).^27^ This was tested for the mortality risk score by itself, and for the mortality risk score combined with the patients age and prefracture independence in ADL. The AUC was interpreted as follows: 0.9-1.0, excellent; 0.8-0.9, good; 0.7-0.8, fair; 0.6-0.7, poor; 0.5-0.6, fail.^28^ Survival analyses were performed to assess the survival and recovery of patients for patients grouped into each tertile of the mortality risk score. Based on these outcomes, a potential cut-off value was explored using regression analyses for having a favorable survival outcome and recovery outcome. A p-value of <0.05 was considered statistically significant for all outcomes. All statistical analyses were performed using IBM SPSS statistics PC software version 25.0. The raw data and the analyses are available upon request.

## Results

Complete data on the metabolomics and the characteristics age, sex, general health status, cognitive status and prefracture living situation were available from all patients. The remaining characteristics (prefracture mobility, independence in ADL and nutritional status) were complete for 126 (92.0%) patients.

The mean age of all patients was 82.1 years and the majority (68.5%) were female. Treatment was performed with an arthroplasty in 44.5%, with internal fixation in 53.3% and conservatively for 2.2% of patients.

Significant differences were observed for all baseline characteristics between patients who did and did not survive during follow-up, except sex and fracture type (Table 1). Of the metabolites, only S-HDL-L, VLDL-D, albumin and glycoprotein acetyls had different means for each group. There was a significant difference in the mean mortality risk score for patients who did and did not survive during follow-up (−0.097; SD, 0.62 and 0.42; SD, 0.87 respectively, P = 0.001). The distribution of the mortality risk score for each group (those who did and did not survive) is presented in Appendix B.

**Table 1.** Baseline Characteristics and Metabolic Profile for Patients With a Proximal Femoral Fracture.

Characteristic	Alive, N = 111 (81.0%)	Dead, N = 26 (19.0%)	P-value
**Patient characteristic**			
Age, y (SD)	81.0 (9.7)	86.8 (7.9)	0.005
Sex, f (%)	76 (68.5)	16 (61.5)	0.50
ASA classification (%)			
I-II	53 (47.7)	3 (11.5)	
III-V	58 (52.3)	23 (88.5)	0.001
Parker mobility score (%)			
7-9	57 (51.4)	2 (8.0)	
4-6	33 (29.7)	13 (52.0)	
0-3	21 (18.9)	10 (40.0)	<0.001
**Katz ADL score (%)**			
0-1	71 (64.0)	6 (25.0)	
2-3	16 (14.4)	6 (25.0)	
4-6	24 (21.6)	12 (50.0)	0.002
Cognitive impairment (%)	34 (30.6)	15 (57.7)	0.010
Malnourished (%)	46 (44.7)	17 (73.9)	0.011
Living situation (%)			
Independent	61 (55.0)	5 (19.2)	
Homecare or residential home	26 (23.4)	13 (50.0)	
Nursing home	24 (21.6)	8 (30.8)	0.003
Fracture type			
Femoral neck	62 (55.9)	12 (46.2)	
(Sub)trochanteric	49 (44.1)	14 (53.8)	0.372
Metabolic profiling*			
XXL-VLDL-L	0.18 (0.17)	0.16 (0.18)	0.63
S-HDL-L	1.08 (0.19)	0.93 (0.17)	<0.001
VLDL-D (nm)	37.97 (1.29)	37.41 (1.20)	0.045
PUFA/FA (%)	41.13 (3.01)	40.74 (3.56)	0.56
Glucose	6.66 (1.93)	6.61 (2.25)	0.91
Lactate	2.07 (0.88)	2.30 (0.85)	0.23
Histidine	0.06 (0.01)	0.06 (0.01)	0.74
Isoleucine	0.05 (0.02)	0.05 (0.02)	0.42
Leucine	0.09 (0.03)	0.08 (0.02)	0.66
Valine	0.20 (0.04)	0.19 (0.04)	0.13
Phenylalanine	0.05 (0.02)	0.06 (0.01)	0.36
Acetoacetate	0.08 (0.10)	0.07 (0.09)	0.56
Albumin (g/l)	35.77 (3.78)	33.38 (5.17)	0.008
Glycoprotein acetyls	0.88 (0.16)	0.97 (0.25)	0.019
Mortality risk score	-0.097 (0.62)	0.42 (0.87)	0.001

SD standard deviation, f female, ASA American Society of Anesthesiologists, ADL activities of daily living. * Means, concentrations are presented in millimole per liter (mmol/l) unless stated otherwise. nm nanometer, g/l gram per liter, XXL-VLDL-L Total lipids in chylomicrons and extremely large VLDL, S-HDL-L total lipids in small HDL, VLDL-D mean diameter for VLDL particles, PUFA/FA ratio of polyunsaturated fatty acids to total fatty acids. Italics indicate a P-value <0.05.

The median follow-up was 6 months (IQR 6) and 26 (19.0%) patients died within this period. The calculated mortality risk score ranged between −1.36 and 2.26.

### Mortality

**Figure 1.**
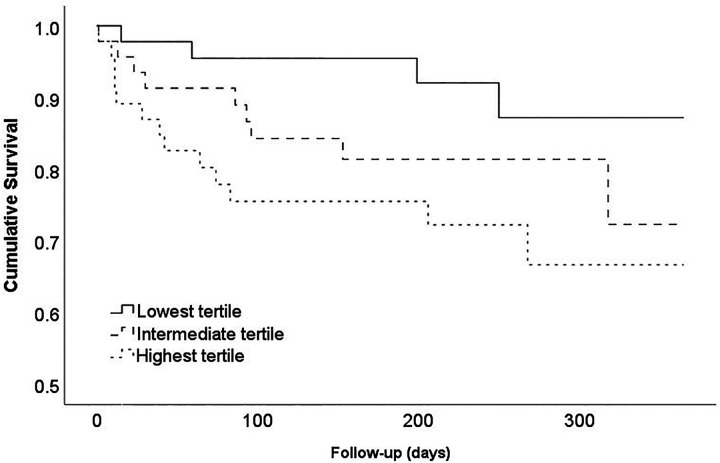
Survival curve of the overall survival stratified for the mortality risk score tertiles. P = 0.049.

For every unit increase in this score, a 2.74 times higher mortality risk was observed in this cohort (HR, 2.74; 95% CI, 1.61-4.66; P < 0.001). The survival analysis indicates a 19.6% difference in the 1-year survival rate between patients from the highest and lowest tertiles, which was borderline significant (P = 0.049; Figure 1). The biggest difference was observed between patients from the lowest tertile versus the medium and highest tertiles. This potential cut-off value (a mortality risk score of ≥−0.4055 or <−0.4055) yields a statistically significant hazard ratio of 2.99 (95% CI, 1.03-8.68; P = 0.044). The mortality risk score by itself showed a fair prediction accuracy for mortality (AUC = 0.68; 95% CI, 0.56-0.81). The model was enhanced to a good level of prediction accuracy when the mortality risk score was combined with the factors age and prefracture independence in ADL (AUC = 0.78; 95% CI, 0.68-0.88).

When results were stratified for fracture type, similar results were observed for both femoral neck fractures (HR, 2.88; 95% CI 1.23-6.74; P = 0.015; AUC = 0.72, Appendix Figure C1a) and (sub)trochanteric fractures (HR, 2.61; 95% CI, 1.28-5.33; P = 0.009; AUC = 0.63, Appendix Figure C1b).

### Recovery

**Figure 2.**
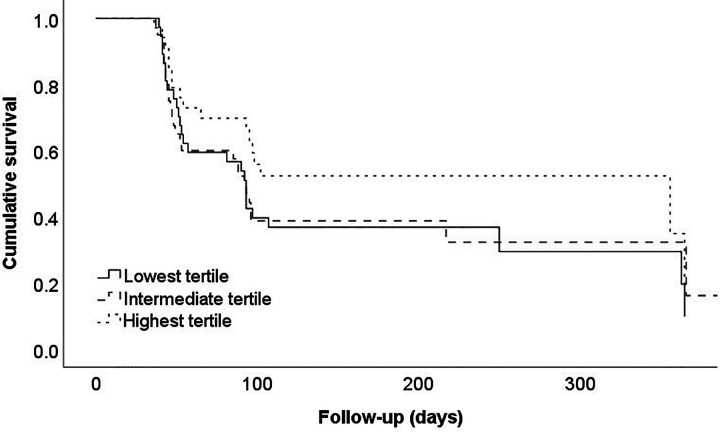
Survival curve of the functional recovery stratified for the mortality risk score tertiles. P = 0.31. Here, an event is defined as a patient recovering to their individual prefracture level of independence for activities of daily living.

Data on the independence in ADL was available for 132 (96.4%) patients. Of these, 70 (51.1%) recovered to their individual prefracture level of independence in ADL. No significant association was found between the risk of not recovering and the mortality risk score (HR, 0.72; 95% CI, 0.47-1.10; P = 0.1283) and although a 25.6% difference was observed in the recovery rate between patients from the highest and lowest tertiles, this was not statistically significant (P = 0.31; Figure 2). Applying the potential cut-off value of the mortality risk score (≥−0.4055 or <−0.4055) to the functional recovery outcomes of patients yields no statistically significant hazard ratio (HR, 0.72; 95% CI, 0.47-1.10; P = 0.128). The tested predictive accuracy of the mortality score by itself indicated an AUC of 0.63 (fair) which enhanced after inclusion of the factors age and prefracture independence in ADL (AUC = 0.67; 95% CI, 0.58-0.76).

No significant results were observed when outcomes were stratified for the fracture types: femoral neck fractures (HR, 1.02; 95% CI 0.53-1.95; P = 0.95; AUC = 0.56, Appendix Figure C2a) and (sub)trochanteric fractures (HR, 0.599; 95% CI 0.34-1.06; P = 0.077; AUC = 0.67, Appendix Figure C2b).

## Discussion

This preliminary study investigates the potential value of metabolomic profiling using a mortality risk score based on 14 metabolites to establish the mortality risk and recovery capacity of patients with proximal femoral fractures. These 14 metabolites have been associated previously with mortality in a range of EU population-based cohorts. A significant association with overall mortality and a borderline significant difference in the mortality rate of each tertile was found for the mortality risk score. The 2.74 times increased risk for mortality per unit increment of the mortality risk score corresponds neatly with the one found in the study of 11 EU cohorts (N = 44.000) by Deelen et al. (HR = 2.73).^3^ In this study the predictive power of the score constructed on the basis of one (Estonian) study was validated in another (Finnish) cohort. In the validation, the AUC of the 5- and 10-year mortality was 0.84 and 0.83 respectively, which proved more effective than the predictive accuracy of models using conventional risk factors.^1,3^ The fair predictive accuracy of the mortality risk score reached in this study was much lower, but improved slightly when the conventional risk factors age and prefracture independence in ADL were added. The significant association between the mortality risk score and survival implies that metabolic profiling could potentially contribute to the prognostic accuracy in a model that combines both metabolomics and patient characteristics. The univariate study of each metabolite separately indicated that especially small HDL levels, the mean diameter for VLDL particles, albumin and glycoproteins differed significantly between the 2 groups. These markers have also been associated with cardiometabolic health and systemic inflammation. Small HDL and the mean diameter for VLDL particles are involved with lipid metabolism, and their regulation of plasma triglyceride is a potential risk factor for mortality.^29^ Albumin and glycoprotein acetyls play an important role in inflammation.^30,31^ Although roles between the other metabolites included in the risk score and health have been described previously, their association with mortality could be explored more in-depth in future studies.^3^

The mortality risk score showed a more reserved association with a fair predictive accuracy for the recovery of independence in ADL. Although some of the metabolites in the score are related to the nutritional status, evidence on the relevance of the nutritional status and functional recovery in patients with proximal femoral fractures is limited.^20^ Biomarker corresponding with functional outcome would represent the physical capacity to recover, which could be a construct of muscle status, endurance performance and metabolic health. However, aspects such as social support and self-determination might also play significant roles. Few studies have investigated these, possibly because they are harder to objectify than many other factors.^32^

### Strengths and Limitations

To our knowledge, this is the first study to attempt metabolic profiling in patients with a proximal femoral fracture.

Based on the patient characteristics this cohort seems representative of the average patient population. The number of patients included in this study was limited, and this sample size restricted the types of analyses that could be performed. As such, only age and prefracture functionality were added as covariates in multivariate models, purely to observe their effect on the accuracy of the model. A larger study with more patients should be used to validate the independent value of the mortality risk score, and could validate the findings of this preliminary study. Extensive validation should also be performed on the proposed cut-off value if it were to be applied in clinical practice.

The mortality risk score that was used in this study was designed to predict long-term survival in general populations. There are substantial differences between that population and the patients with a proximal femoral fracture. Patients with a proximal femoral fracture are exposed to significant excess mortality risks, with 1-year mortality rates between 20-25%.^7-9^ This is substantially higher than the 12.5% mortality rate within the 2.76 years follow of the previous study by Deelen et al. in European cohort studies.^3^ A risk score based on metabolites tailored for the patient population with a proximal femoral fracture only, could in theory be more effective. However, developing this would require a substantially larger number of patients.

The set of metabolites included in this study and all those investigated by Deelen et al., form only a fraction of all available metabolites in the human serum.^2,3^ Other sets of metabolites which have not yet been studied for these purposes could also prove more effective in predicting patient outcomes.

## Conclusion

Although a modest prediction accuracy was observed for the mortality risk score in this population compared to those previously studied, the metabolomic profile assessed in this preliminary study is significantly associated with survival and aspects of it can potentially improve the prognostic accuracy for patients with a proximal femoral fracture. More elaborate studies are needed to develop a comprehensive model for clinical purposes.

## Appendix A

The mortality score as described by Deelen et al. was based on a study of the same metabolite platform as the current study but applied to 44.000 individuals indicating 136 highly correlated biomarkers out of 226 to be associated with mortality. A stepwise forward-backward regression procedure on the 63 (out of 226) least correlated markers revealed that 14 metabolites independently and significantly associated with mortality. The mortality score in the current paper, based on these 14 sampled metabolites, was calculated for each individual patient according to the procedure by Deelen et al. This requires summing the weighted metabolites after log-transformation and scaling them: Mortality risk score = (((Zln[XXL_VLDL_L])*ln(0.80)) + ((Zln[S_HDL_L])*ln(0.87)) + ((Zln[VLDL_size])*ln(0.85)) + ((Zln[PUFA_FA])*ln(0.78)) + ((Zln[Glucose])*ln(1.16)) + ((Zln[Lactate])*ln(1.06)) + ((Zln[his])*ln(0.93)) + ((Zln[Ile])*ln(1.23)) + ((Zln[Leu])*ln(0.82)) + ((Zln[Val])*ln(0.87)) + ((Zln[Phe])*ln(1.13)) + ((Zln[Acetoacetate])*ln(1.08)) + ((Zln[Albumin])*ln(0.89)) + ((Zln[GlycA])*ln(1.32))).

## Appendix B

The distribution of the mortality risk score for patients who did and did not survive. Mean score for no mortality: −0.097 (standard deviation, 0.62; range −1.36 to 1.67) and mortality 0.42 (standard deviation, 0.87; range −1.25 to 2.26), P = 0.001.

## Appendix C

Survival curves of the overall survival and functional recovery, stratified for the mortality risk score tertiles and for each fracture type. (1a) The overall survival for femoral neck fracture patients. N = 63, P = 0.067. (1b) The overall survival for (sub)trochanteric fracture patients. N = 74, P = 0.59. (2a) The functional recovery for femoral neck fracture patients. N = 61, P = 0.93. (2b) The functional recovery for (sub)trochanteric fracture patients. N = 71, P = 0.33.
